# Activated prothrombin complex concentrate to reverse the factor Xa inhibitor (apixaban) effect before emergency surgery: a case series

**DOI:** 10.1186/s13256-018-1660-9

**Published:** 2018-05-16

**Authors:** Nina Haagenrud Schultz, Runar Lundblad, Pål Andre Holme

**Affiliations:** 1 Department of Haematology, Oslo University Hospital, Box 4950, N-0424 Oslo, Norway; 2 Research Institute of Internal Medicine, Oslo University Hospital, Box 4950, N-0424 Oslo, Norway; 3 Institute of Clinical Medicine, University of Oslo, Box 1171, Blindern, N-0318 Oslo, Norway; 4 Department of Haematology, Akershus University Hospital, N-1478 Lørenskog, Norway; 5 Department of Cardiothoracic Surgery, Oslo University Hospital, Box 4950, N-0424 Oslo, Norway

**Keywords:** FXa inhibitors, Apixaban, Emergency surgery, Reversal, Activated prothrombin complex concentrate

## Abstract

**Background:**

The lack of an antidote against factor Xa inhibitors in case of major bleeding or need for urgent surgery is a concern to clinicians. Guidelines on managing major bleeding in patients under anticoagulation with a factor Xa inhibitor suggest several hemostatic agents to reverse the effect, but there is no consensus regarding the choice of drug or appropriate dose. The ability of prothrombin complex concentrate, activated prothrombin complex concentrate, and recombinant factor VIIa to reverse the effect of factor Xa inhibitors has been evaluated in animal studies, *in vitro* studies, and healthy volunteers, but not yet in randomized clinical studies.

**Case presentation:**

We report a consecutive case series of patients under factor Xa inhibitor (apixaban) treatment who received activated prothrombin complex concentrate to reverse the anticoagulation effect before emergency cardiovascular surgery. Patient 1, a 63-year-old white man, was operated with replacement of the aortic valve; patient 2, a 65-year-old white man, underwent heart transplantation; patient 3, a 68-year-old white man, was operated for acute type A aortic dissection. They all received activated prothrombin complex concentrate 25 IU/kg immediately before surgery. In two of the cases, the global coagulation assay thromboelastometry (ROTEM™) was performed before and after administering activated prothrombin complex concentrate. The ROTEM™ clotting time was reduced from 1900 seconds to 740 seconds and from 1482 to 807 seconds, respectively, after administering a dose of 25 IU/kg activated prothrombin complex concentrate. The apixaban concentration before reversal was within the range considered to be the therapeutic level in all cases. No bleeding complications occurred during surgery, but one case was complicated with bleeding postoperatively. No thromboembolic complications were observed during or after surgery.

**Conclusions:**

Activated prothrombin complex concentrate 25 IU/kg reversed the anticoagulation effect of apixaban effectively and safely before emergency cardiovascular surgery.

## Background

Factor Xa (FXa) inhibitors are direct oral anticoagulants (DOACs) and safe and effective alternatives to warfarin in preventing stroke in patients with atrial fibrillation and treating venous thrombosis [1]. Apixaban (Eliquis©; Bristol-Myers Squibb/Pfizer EEIG, Uxbridge, United Kingdom) is an oral, direct-acting FXa inhibitor with a half-life of approximately 12 hours. The treatment is less complicated than warfarin treatment, as it consists of a fixed dose which does not require monitoring [2]. Because of the convenience and proven efficacy, the prescription rate of DOACs is increasing worldwide [3]. Consequently, the number of patients who are anticoagulated with DOACs and need emergency surgery for coexisting conditions increases accordingly. However, reversal of the effect of FXa inhibitors is still a matter of debate. The antidote andexanet alfa has shown promising results in clinical studies, but it is still not commercially available [4]. Furthermore, this antidote is not a good option for reversing the FXa inhibitor effect before cardiovascular surgery, as it is unspecific and reverses all FX inhibitors, including heparinization, that are required during cardiopulmonary bypass. Guidelines providing a strategy to manage bleeding complications or emergency surgery in FXa inhibitor-treated patients are not conclusive and suggest 4-factor prothrombin complex concentrate (4-PCC) or activated prothrombin complex concentrate (aPCC) as reversal agents [5]. 4-PCC contains factor II, VII, IX, and X in their inactive zymogen forms and is used to reverse the anticoagulation effect of warfarin. aPCC is used to treat hemophiliacs with inhibitor and contains activated FVII in addition to factor II, IX, and X. The documentation of the ability of these reversal agents to reverse the anticoagulation of FXa inhibitors is limited, and apart from one cohort study evaluating the effect of 4-PCC as reversal agent in apixaban-treated patients undergoing major bleeding [6], only animal studies, *in vitro* studies, and case reports exist [7–12]. In the following case series we present three patients in whom aPCC was used to reverse the anticoagulation effect of apixaban before emergency cardiovascular surgery. The reversal effect was assessed both clinically by the surgeon and by coagulation tests.

## Case presentation

### Case 1

**Fig. 1:**
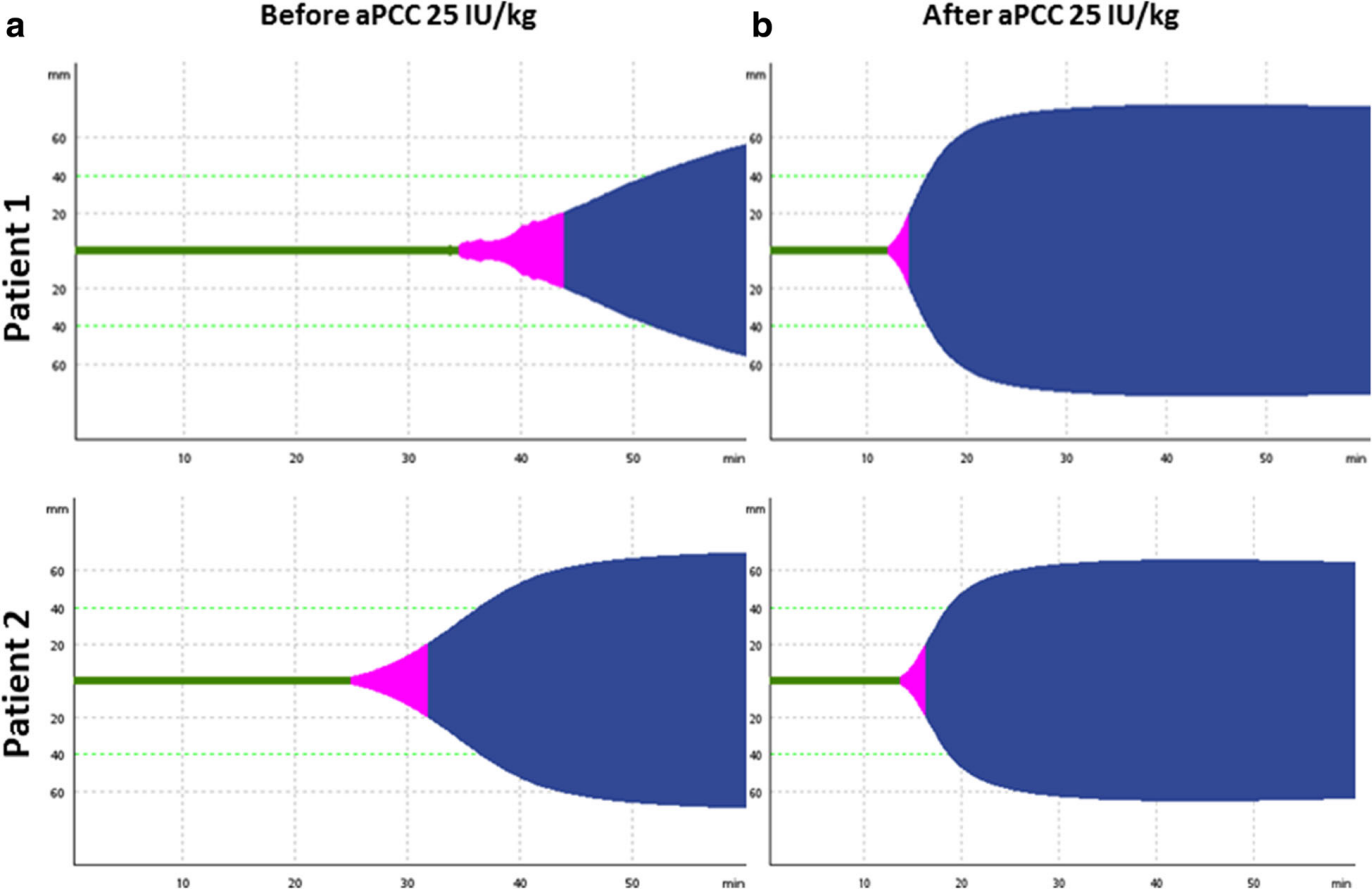
Thromboelastometry curves of patient 1 and patient 2. **a** Thromboelastometry curves before administering activated prothrombin complex concentrate 3000 IU. **b** Thromboelastometry curves after administering activated prothrombin complex concentrate 3000 IU. Clotting time shortened after administering activated prothrombin complex concentrate 3000 IU. *aPCC* activated prothrombin complex concentrate

A 63-year-old white man under treatment with apixaban 5 mg twice daily due to atrial fibrillation was hospitalized after rapidly developing symptoms of heart failure. Four weeks earlier he had had a re-implantation of a biological aortic valve because of infectious deposits on the mechanical valve and was still under antibiotic treatment at the time of admission. He had no previous history of bleeding disorders. He had taken his morning dose of apixaban and presented with respiratory distress, fever, and hypotension. His blood samples showed a hemoglobin level of 97 g/L, leukocyte count of 10.3 × 10^9^/L, thrombocyte count of 157 × 10^9^/L, estimated glomerular filtration rate (GFR) of 45 ml/minute, and C-reactive protein concentration of 337 mg/L. He had normal liver function tests. Both prothrombin time (PT) and activated partial thromboplastin time (aPTT) were prolonged (Table 1). An echocardiography revealed an extreme aorta stenosis and a left ventricle dysfunction. His condition deteriorated rapidly, and surgery to replace the aortic valve was needed immediately. There was no time to await the wash-out effect of apixaban. Due to recent apixaban tablet intake and need for major surgery with potentially large blood loss, aPCC (FEIBA^©^) 3000 IU (25 IU/kg) was administered over a 10-minute period prior to surgery to reverse the anticoagulation effect of apixaban. Afterwards, cardiopulmonary bypass was established with full heparinization, which was monitored with aPTT. Before and after the aPCC treatment, but before starting the heparin infusion, blood samples were collected in citrated test tubes prefilled with corn trypsin inhibitor (CTI) and in test tubes containing only citrate. The apixaban concentration was measured by anti-FXa activity (aFXa) assay, and coagulation status was assessed by thromboelastometry (ROTEM®; Tem Innovations, Munich, Germany) using minimal tissue factor activation [13, 14], PT, and aPTT before and after aPCC treatment. ROTEM™ clotting time (CT) was shortened from 1900 to 740 seconds, and clot formation time (CFT) was shortened from 353 to 191 seconds. PT and aPTT were reduced from 62 to 20 and 58 to 48, respectively. Surgery was performed successfully without excessive bleeding or thromboembolic complications. Hence, administering aPCC improved hemostasis, which was assessed clinically by the surgeon and measured by global coagulation tests (Fig. 1a, b) (Table 1).

**Table 1:** Laboratory results of patients 1 and 2 before and after reversal of the apixaban effect

	Case 1		Case 2	
	Before aPCC	After aPCC	Before aPCC	After aPCC
CT (seconds)	1900	740	1482	807
MCF (mm)	60	55	68	66
CFT (seconds)	353	191	353	159
PT (seconds)	62	20	26	< 17
aPTT (seconds)	58	48	33	27
aFXa (μg/L)	69	68	152	152

*aFXa* anti-factor Xa activity, *aPCC* activated prothrombin complex concentrate, *aPTT* activated partial prothrombin time, *CFT* clot formation time, *CT* clotting time, *MCF* maximum clot firmness, *PT* prothrombin time

### Case 2

A 65-year-old white man waiting for heart transplantation because of end-stage heart failure was under treatment with apixaban 5 mg twice daily because of atrial fibrillation. He had no other comorbidities and had no previous history of bleeding disorders. On the day a heart from a deceased donor was available, he had taken his morning dose of apixaban. Heart transplantation surgery was coordinated the same evening. His blood test results at the time of admission showed normal blood counts, creatinine level of 156 μmol/L, estimated GFR 40 ml/minute, PT 26 seconds and aPTT 33 seconds. The apixaban concentration was 152 μg/L. Because of the recent tablet intake and the great bleeding risk during and after the procedure, it was decided to administer aPCC 3000 IU immediately before surgery. His body weight was 108 kg, which means that the dose administered was 28 IU/kg. The surgery was uncomplicated, and so was the postoperative period. No bleeding complications or thromboembolic events were seen. CT was reduced by more than 50% (from 1482 seconds to 807 seconds), and CFT was reduced accordingly (Fig. 1a, b). PT and aPTT were also reduced after aPCC treatment (Table 1).

### Case 3

A 68-year-old white man taking apixaban 5 mg twice daily because of atrial fibrillation was hospitalized with acute chest pain. He had no other comorbidities and no prior bleeding disorders in the past. A quickly performed computed tomography scan showed a type A aortic dissection, and acute surgery was required. He had taken the last dose of apixaban the same morning, 8 hours earlier. He had normal kidney function at the time of admission. For the same reasons as in the previous cases, it was decided to administer aPCC 3000 IU (29 IU/kg) prior to surgery. The surgery went well without excessive bleeding, but after 4 hours postoperative pericardial bleeding occurred, causing tamponade. He was re-operated twice the same night because of bleeding, and he received massive transfusion with erythrocytes, thrombocytes, plasma, and, later, prothrombin complex concentrate, fibrinogen, aPCC, and recombinant activated FVII. By the next morning, approximately 8 hours later, he stopped bleeding and was stabilized. Unfortunately, the blood samples were contaminated with heparin, so there are no ROTEM™ results in this case. The apixaban concentration was 98 μg/L, but this value was not known at the time of surgery and did not influence the treatment before or after surgery. Postoperatively, no thromboembolic complications were observed, but he died of septic shock after 6 weeks in the intensive care unit.

## Discussion

The optimal method to reverse the effect of FXa inhibitors in cases of emergency surgery or major bleeding is not known. In this case series, we report on the cases of three patients anticoagulated with apixaban who required emergency cardiovascular surgery. By administering aPCC prior to surgery, the anticoagulation effect was reversed successfully, according to measurements of global coagulation assays and clinical assessment.

The therapeutic level of apixaban is not yet established, but aFXa in patients anticoagulated with apixaban 5 mg twice daily have peak values of 91 to 321 μg/L and trough values of 41 to 230 μg/L [2]. In the cases reported here, the aFXa level calibrated for apixaban was between 68 and 152 μg/L at the time of admission, which confirms that the patients were fully anticoagulated.

Cardiovascular surgery has a high risk of bleeding complications and involves cardiopulmonary bypass treatment, including full heparinization. For this reason it was decided to reverse the anticoagulation effect of apixaban. In the acute setting, aPCC 3000 IU (25-30 IU/kg) was administered over 10 minutes immediately before initiating heparinization and cardiopulmonary bypass treatment to perform major cardiovascular surgery. This dose was chosen to minimize the risk of thromboembolic complications. *In vitro* studies have shown that aPCC at the suggested dose might overcorrect the hemostatic parameters [7, 9], and patients treated with apixaban already have a risk of thromboembolic complications. Another study has shown that 25 IU/kg reverses the hemostatic parameters altered by apixaban quickly and sufficiently [10]. A single case report also supports the use of this dose [15].

In addition to a clinical assessment of hemostasis, the reversal effect of aPCC was measured by thromboelastometry, which provides information about time until clot formation and the viscoelastic properties of the clot. Previous studies performed with the same method have shown that the healthy, not anticoagulated control group obtained an average CT of 705 seconds (± 210 seconds) [9]. This means that the reported cases of patients obtained a CT corresponding to normal value after receiving aPCC. AFXa activity was, not surprisingly, unaltered after aPCC treatment, as the activated clotting factors improve the hemostasis by bypassing the FXa inhibition of apixaban but do not directly influence the apixaban concentration. The fact that the conventional coagulation tests PT and aPTT are not valuable indicators of the DOAC effect [16] was confirmed in this case series. In cases 2 and 3 the values of aPTT were within the normal range before reversal of the apixaban effect, but in case 1, aPTT was higher than expected when taking the apixaban concentration in consideration. We do not know the reason for this, but one possible explanation may be that there was a situation with disseminated intravascular coagulation, as the patient had an infection at time of admission. In patient 1, the concentration of apixaban was surprisingly high 24 hours after last intake, which may be explained by the impaired kidney function.

The main limitation of this case series is that only a few cases are described. The risk of selection bias, however, is reduced by consecutively including patients. The lack of a control group is another limitation; however, the patients served as their own controls because laboratory tests were performed before and after treatment. In two of three patients, the reversal was successful, and this may also have been the case in the third patient. The postoperative bleeding could have been caused by an arterial bleeding, and reversing the anticoagulation effect would not have prevented this. The case report only described the use of one of the suggested treatment options, and randomized studies are needed to provide us with the optimal treatment in cases of major bleeding or the need for emergency cardiovascular surgery in FXa inhibitor-treated patients.

## Conclusions

This case series describes the use of aPCC in a setting where reversal of the anticoagulation effect of apixaban was needed before acute cardiovascular surgery. Our report demonstrates that hemostasis markedly improved, as assessed clinically and by coagulation tests after administering aPCC 25-29 IU/kg. None of these surgeries was complicated by excessive bleeding during surgery. However, one of the cases was complicated by postoperative bleeding. The safety of the treatment was demonstrated, as in none of the cases did thromboembolic complications occur (Table 2).

**Table 2:** Apixaban concentration, treatment, and clinical course

	aFXa (μg/L)	Time from last apixaban dose to surgery	aPCC dose	Excessive perioperative or postoperative bleeding	Thromboembolic complications
Patient 1	69	24 hours	25 IU/kg	No	No
Patient 2	152	10 hours	28 IU/kg	No	No
Patient 3	98	9 hours	29 IU/kg	Yes	No

*aFXa* anti-factor Xa activity, *aPCC* activated prothrombin complex concentrate

## Abbreviations

4-PCC: 4-factor prothrombin complex concentrate
aFXa: Anti-factor Xa activity
aPCC: Activated prothrombin complex concentrate
aPTT: Activated partial thromboplastin time
CFT: Clot formation time
CT: Clotting time
CTI: Corn trypsin inhibitor
DOAC: Direct oral anticoagulant
FXa: Factor Xa
GFR: Glomerular filtration rate
PT: Prothrombin time

## References

[1] van Es N, Coppens M, Schulman S, Middeldorp S, Buller HR. Direct oral anticoagulants compared with vitamin K antagonists for acute venous thromboembolism: evidence from phase 3 trials. Blood. 2014;124(12):1968–75. 10.1182/blood-2014-04-571232 24963045

[2] Eikelboom JW, Quinlan DJ, Hirsh J, Connolly SJ, Weitz JI. Laboratory monitoring of non-vitamin K antagonist oral anticoagulant use in patients with atrial fibrillation: a review. JAMA Cardiol. 2017;2:566–74. 10.1001/jamacardio.2017.0364 28355459

[3] Loo SY, Dell'Aniello S, Huiart L, Renoux C. Trends in the prescription of novel oral anticoagulants in UK primary care. Br J Clin Pharmacol. 2017;83(9):2096–106. 10.1111/bcp.13299 PMC5555878 28390065

[4] Siegal DM, Curnutte JT, Connolly SJ, Lu G, Conley PB, Wiens BL, et al. Andexanet Alfa for the Reversal of Factor Xa Inhibitor Activity. N Engl J Med. 2015;373(25):2413–24. 10.1056/NEJMoa1510991 26559317

[5] Faraoni D, Levy JH, Albaladejo P, Samama CM. Updates in the perioperative and emergency management of non-vitamin K antagonist oral anticoagulants. Crit Care. 2015;19(1):203. 10.1186/s13054-015-0930-9 PMC4414429 25925382

[6] Majeed A, Ågren A, Holmström M, Bruzelius M, Chaireti R, Odeberg J, et al. Management of rivaroxaban- or apixaban-associated major bleeding with prothrombin complex concentrates: a cohort study. Blood. 2017;130(15):1706–12. 10.1182/blood-2017-05-782060 28835439

[7] Marlu R, Hodaj E, Paris A, Albaladejo P, Cracowski JL, Pernod G. Effect of non-specific reversal agents on anticoagulant activity of dabigatran and rivaroxaban: a randomised crossover *ex vivo* study in healthy volunteers. Thromb Haemost. 2012;108(2):217–24. 10.1160/TH12-03-0179 22627883

[8] Escolar G, Fernandez-Gallego V, Arellano-Rodrigo E, Roquer JC, Reverter JC, Sanz VV, Molina P, Lopez-Vilchez I, Diaz-Ricart M, Galan AM. Reversal of apixaban induced alterations in hemostasis by different coagulation factor concentrates: significance of studies *in vitro* with circulating human blood. PLoS One. 2013;8:e78696. 10.1371/journal.pone.0078696 PMC3823858 24244342

[9] Schultz NH, Tran HT, Bjornsen S, Henriksson CE, Sandset PM, Holme PA. The reversal effect of prothrombin complex concentrate (PCC), activated PCC and recombinant activated factor VII against anticoagulation of Xa inhibitor. Thromb J. 2017;15:6. 10.1186/s12959-017-0129-1 PMC5319105 28239301

[10] Schultz NH, Tran HTT, Bjørnsen S, Henriksson CE, Sandset PM, Holme PA. The reversal effect of prothrombin complex concentrate (PCC), activated PCC and recombinant activated factor VII in apixaban-treated patients *in vitro*. Research and Practice in. Thromb Haemost. 2017;1:49–56. 10.1002/rth2.12015 PMC6058213 30046673

[11] Martin AC, Gouin-Thibault I, Siquret V, Mordohay A, Samama CM, Gaussem P, Le Bonniec B, Godier A. Multimodal assessment of non-specific hemostatic agents for apixaban reversal. J Thromb Haemost. 2015;13:426–36. 10.1111/jth.12830 25630710

[12] Eerenberg ES, Kamphuisen PW, Sijpkens MK, Meijers JC, Buller HR, Levi M. Reversal of rivaroxaban and dabigatran by prothrombin complex concentrate: a randomized, placebo-controlled, crossover study in healthy subjects. Circulation. 2011;124:1573–9. 10.1161/CIRCULATIONAHA.111.029017 21900088

[13] Sorensen B, Johansen P, Christiansen K, Woelke M, Ingerslev J. Whole blood coagulation thrombelastographic profiles employing minimal tissue factor activation. J Thromb Haemost. 2003;1(3):551–8. 10.1046/j.1538-7836.2003.00075.x 12871465

[14] Adelmann D, Wiegele M, Wohlgemuth RK, Koch S, Frantal S, Quehenberger P, et al. Measuring the activity of apixaban and rivaroxaban with rotational thrombelastometry. Thromb Res. 2014;134(4):918–23. 10.1016/j.thromres.2014.08.006 25179518

[15] Rinehart DR, Lockhart NR, Hamilton LA, Langdon JR, Rowe AS. Management of apixaban-associated subdural hematoma: a case report on the use of factor eight inhibitor bypassing activity. Crit Care Med. 2015;43(6):e203–7. 10.1097/CCM.0000000000000909 25738855

[16] Douxfils J, Chatelain C, Chatelain B, Dogne JM, Mullier F. Impact of apixaban on routine and specific coagulation assays: a practical laboratory guide. Thromb Haemost. 2013;110(2):283–94. 10.1160/TH12-12-0898 23765180

